# Comparative analysis of cultivated and wild olive genotypes to salinity and drought stress

**DOI:** 10.3389/fpls.2024.1423761

**Published:** 2024-07-16

**Authors:** Josip Tadić, Gvozden Dumičić, Maja Veršić Bratinčević, Sandra Vitko, Zlatko Liber, Sandra Radić Brkanac

**Affiliations:** ^1^ Department of Plant Sciences, Institute for Adriatic Crops and Karst Reclamation, Split, Croatia; ^2^ Centre of Excellence for Biodiversity and Molecular Plant Breeding (CoE CroPBioDiv), Zagreb, Croatia; ^3^ Department of Applied Sciences, Institute for Adriatic Crops and Karst Reclamation, Split, Croatia; ^4^ Division of Botany, Department of Biology, Faculty of Science, University of Zagreb, Zagreb, Croatia

**Keywords:** *Olea europaea*, ion content, biochemical changes, abiotic stress, wild olive tree

## Abstract

The Mediterranean region’s harsh conditions, characterized by low rainfall, high solar radiation, and elevated temperatures, pose challenges for vegetation, particularly in the face of climate change. Cultivated olive (*Olea europaea* subsp. *europaea* var. *europaea*) holds historical and economic significance as one of the oldest crops in the Mediterranean. Due to their high germplasm diversity and greater flowering abundance compared to the offspring of cultivated olives, wild olives (*Olea europaea* subsp. *europaea* var. *sylvestris*) could be utilized for selecting new olive cultivars capable of adapting to a changing climate. This research aimed to compare the effects of salt and drought stress on wild and cultivated genotypes by analyzing morphological, physiological, and biochemical parameters. Results showed that shoot length, shoot dry mass, and leaf area are key drought stress indicators in wild olive trees. The results indicated the olive trees more susceptible to salinity stress had lower Na^+^ and Cl^-^ concentrations in their leaves and took longer to stabilize salt ion levels. Decreased K^+^ content in roots across all treatments indicated a general stress response. The uptake of Ca^2+^ appears to be the most energy-efficient response of olive trees to short-term salinity and drought. In contrast to proline and malondialdehyde, trends in superoxide dismutase activity suggest that it is a reliable indicator of salinity and drought stress. Regarding olive adaptability to salinity stress, promising results obtained with two wild olive genotypes merit their further physiological study.

## 1 Introduction

The Mediterranean region is characterized by low rainfall, high solar radiation, and high temperatures, which makes the vegetation in this area more susceptible to climate changes associated with drought (Brito et al., 2019). Droughts are becoming more frequent due to increased interannual variability in precipitation and longer periods of low rainfall (Gouveia et al., 2017). Climate monitoring models confirm a significant rise in drought events, largely attributed to human-driven greenhouse gas emissions (Hoerling et al., 2012; Gouveia et al., 2017). These issues are already observed in southern Europe and northern Africa, earning the Mediterranean region the label of a climate change “hotspot” (Gao and Giorgi, 2008; Hoerling et al., 2012). Between 1990 and 2010, ten of the driest winters in the past 120 years were recorded in the Mediterranean region (Hoerling et al., 2012). Salinization, the migration of soluble salts into previously salt-free areas, is prevalent in arid and semi-arid regions with low rainfall and coastal areas affected by sea-level rise (Metternicht, 2017; Gould et al., 2021). Natural intrusion of saline or brackish groundwater into freshwater sources can lead to increased soil salinity (Mateo-Sagasta and Jacob, 2011). In irrigated agriculture, the use of low-quality or brackish water is a common practice that contributes to soil salinity (Metternicht, 2017; Harper et al., 2021; Oosterbaan, 2020).

Modern olive cultivation systems, characterized by dense planting in high-density orchards, irrigation, and the use of soluble fertilizers, are gradually replacing traditional methods (De La Rosa et al., 2014). This shift limits the number of cultivars suitable for modern practices and disregards the diversity of local varieties, posing a risk of genetic diversity loss. Clonal selection is hindered by the genetic composition of cultivars, as genetically superior individuals are rare or closely resemble the parent cultivar (Lavee, 2013). Consequently, breeding programs aim to prioritize the identification or development of olive varieties exhibiting distinct traits, such as a shorter juvenile period, less robust growth, and resilience to both abiotic and biotic stresses. Ensuring breeders have access to a wide range of genetic resources diversity is crucial for enhancing, expediting, and optimizing crop improvement methods. However, the constraints imposed by domestication bottlenecks have also limited the diversity within modern breeding populations (Allaby et al., 2019). Abiotic stressors, exemplified by heightened salinity levels and prolonged drought conditions, present escalating challenges within densely planted orchards. This phenomenon becomes particularly pronounced in environments characterized by elevated water requirements, where the utilization of brackish or saline water for irrigation is compounded by the concurrent application of soluble fertilizers (Lavee et al., 2014). Compared to other fruit trees, the olive tree exhibits moderate resistance to salinity and drought, a trait that seems to be cultivar-dependent (Loreto et al., 2003; Chartzoulakis, 2005; Perica et al., 2008; Tattini and Traversi, 2009). Research on wild olives has brought to light agronomically favorable characteristics that are comparatively less noticeable in their cultivated counterparts. Wild olives can exhibit adaptability to diverse environmental conditions (Tadić et al., 2021), excellent regenerative capacity after a fire or frost events, as well as shorter juvenile periods (Baldoni and Belaj, 2009). Due to their high germplasm diversity (Belaj et al., 2011), increased flowering abundance compared to offspring of cultivated olives, wild (*Olea europaea* subsp. *europaea* var. *sylvestris*) and feral olives are valuable reservoirs of genetic material that can be used for selection of new olive cultivars that can adapt to a changing climate (León et al., 2018). However, it is crucial to recognize that wild olive genotypes do not inherently ensure superior plant traits for agricultural purposes. While wild plant relatives may offer genetic diversity and potential traits beneficial for breeding programs, the transfer of desirable traits to cultivated plants is not guaranteed (Dempewolf et al., 2017; Renzi et al., 2022). Additionally, wild relatives may carry undesirable traits or genetic factors that could hinder crop performance or quality, emphasizing the need for thorough evaluation and breeding efforts to harness their potential effectively (Tirnaz et al., 2022).

Under increased salinity and drought, the olive tree experiences osmotic and oxidative stress, and the strength of the stress is determined by the salinity level, more precisely by the concentration of Na^+^ and Cl^-^ ions, as well as the duration of the drought period (Chartzoulakis, 2005; Munns and Tester, 2008). The sclerophyllous leaves of the olive tree have a densely packed mesophyll which can limit the entry of CO_2_ into the chloroplast, thus affecting photosynthesis, although the impact of reduced photosynthetic activity varies depending on the duration of drought and/or exposure to increased salt concentration (Bongi and Loreto, 1989; Tattini et al., 1995; Chartzoulakis et al., 2002). Shoot growth is under greater stress than root growth, similar to drought conditions, and varies from genotype to even the population of the plant species (Chartzoulakis, 2005; Munns and Tester, 2008; Perica et al., 2008; Tattini and Traversi, 2009). Under normal circumstances, plants maintain a high K^+^/Na^+^ ratio in the cell cytosol, while Na^+^ helps maintain osmotic balance in growing tissues (Chartzoulakis, 2005; Munns and Tester, 2008). Under conditions of increased salinity, olive leaves become thicker and more succulent (Bongi and Loreto, 1989). To protect the shoots and young leaves, olive trees preferentially store salt ions in the roots; as salt ions continue to enter, they are transported and accumulate in the stem and older leaves (Chartzoulakis, 2005; Tattini and Traversi, 2009). Olive trees show some tolerance to high Na^+^ and Cl^−^ concentrations in growing tissues by accumulating inorganic ions, mainly K^+^ and Ca^2+^ (Goreta et al., 2007; Perica et al., 2008; Tattini and Traversi, 2009). High Ca^2+^ can also cause greater “osmotic imbalance,” but it restricts the allocation of Na^+^ and Cl^−^ to sensitive shoots and leaves. Olives have an advantage over other fruit crops due to their natural habitat in calcareous soils rich in Ca^2+^ (Tattini and Traversi, 2009).

Besides causing osmotic stress and ionic toxicity, salinity increases the production of reactive oxygen species (ROS), which can damage proteins, DNA, and membrane lipids (Das and Roychoudhury, 2014). To counter oxidative stress, plants have developed a complex antioxidant defense system with enzymatic components like superoxide dismutase (SOD), catalase, ascorbate and guaiacol peroxidase (GPOX), as well as non-enzymatic components such as ascorbic acid, tocopherol, and glutathione (Hasegawa, 2013). Despite the olive tree’s natural adaptation to the unfavorable conditions of the Mediterranean region, salinity and drought continue to limit vegetative growth and yield (Gucci and Caruso, 2011).

We postulated that young wild olive genotypes originating from wild olive trees growing in natural locations along the Adriatic coast might display a greater ability to withstand salt and drought stress than cultivated olive trees. By doing so, the study sought to determine the practical applicability and suitability of different olive genotypes in a changing climate.

Thus, the main objective of this study was to evaluate and compare the effects of short-term yet intense salinity and drought stress on both wild and cultivated olive genotypes. To achieve this aim, we examined the responses of olive trees in the following areas:

1) Growth, by measuring morphological parameters such as shoot length, leaf surface area, and dry weight.

2) Ionic and osmotic relations, by assessing the content of Na, Cl, K, Ca, and Mg ions, as well as proline levels.

3) Oxidative status, by evaluating lipid peroxidation and the activities of the antioxidative enzymes superoxide dismutase (SOD) and guaiacol peroxidase (GPOX).

## 2 Material and methods

### 2.1 Collection and preparation of olive samples for the experiment

Samples of shoot cuttings for vegetative propagation of cultivated olives (*Olea europaea* subsp. *europaea* var. *europaea*) were collected from multivarietal orchards at two olive field collections: Split (43.504678, 16.499206) and Kaštel Stari (43.557061, 16.348383). Those locations are official field collections (germplasm banks) of the Institute for Adriatic Crops and Karst Reclamation, with a Mediterranean climate and eutric brown soil (pH 5.5–6.8, humus 2–6%). Wild olive genotypes (*Olea europaea* subsp. *europaea* var. *sylvestris*) were collected from eight locations along the Adriatic coast where wild olives naturally occur (**Table 1**, **Supplementary Figure 1**). Using a literature review (Zec, 1951; Vlašić, 1980), these wild genotypes were selected based on morphological characteristics such as the smooth surface of the olive stone, significantly smaller leaf and fruit dimensions, as well as the proximity of the olive individuals to old or neglected olive groves. All olive samples used in this study have already been genotyped in previous genetic studies (Klepo, 2014; Tadić et al., 2021; Klepo et al., 2024) and, in this study, they are characterized as wild or cultivated. Shoot cuttings of 10 to 15 cm, were fully immersed in the systemic fungicide Zino (Ningbo Synagrochem Co., Ltd., China). A rooting solution (2,500 ppm) was prepared using redistilled water, 96% ethyl alcohol, and indole-3-butyric acid (Sigma-Aldrich, St. Louis, United States). The basal ends of the cuttings were dipped in the solution for 10 seconds and then allowed to dry completely. Once dried, the cuttings were placed in a mist propagation system on a heated rooting table filled with a layer of perlite. Successfully rooted cuttings were then transplanted into small containers filled with a mixture of Agrilit 3 perlite (Perlite Italiana SRL., Milano, Italy) and Brill TYPical 4 substrate (Brill Substrate GmbH & Co. KG, Georgsdorf, Germany). After an acclimatization period, the young plants were transplanted again into 5 L pots containing a mixture of Brill TYPical 4 substrate, Agrilit 3 perlite, and eutric brown soil in a 2:1:2 ratio by volume and placed in an unshaded greenhouse.

**Table 1:** List of olive genotypes used in the study.

Wild olive genotypes	Cultivated olive genotypes
Sample label	Cultivar
**LA 13**	‘Koroneiki’
**LN 11**	‘Leccino’
**PLJ 18**	‘Oblica’
**MLJ 25**	
**‘Piculja’**	
**PLJ 7**	
**PLJ 22**	

### 2.2 Experimental setup and plant preparation

After a year of growth in the greenhouse, the plants were removed from their pots, cleaned, and transplanted into 3.6 L plastic pots. These pots were filled with a 1:1 mixture of Agrilite 3 perlite and vermiculite (RHP, Gravenzande, the Netherlands). To prevent substrate leakage, 350 ml of expanded clay (Laterlite S.P.A, Milano, Italy) was placed at the bottom of each pot. The acclimatization occurred in the greenhouse under natural light conditions from March to June. The plants were pruned to maintain a single shoot and irrigated daily with half-strength Hoagland nutrient solution (½ HNS) (Hoagland and Arnon, 1950) with an average height of 128 cm. Daily percolate was analyzed to maintain a 20–30% leaching fraction, ensuring proper pH and electrical conductivity. An automatic control system (Schneider Electric, Rueil-Malmaison, France) regulated the irrigation regime for each treatment and controlled the greenhouse temperature through side and roof ventilation. A total of 10 genotypes (7 wild and 3 cultivated genotypes) were selected for morphological and biochemical characterization. Since the focus of the study was on the comparative analysis of wild and cultivated olives, the chosen genotypes were compared with reference cultivars (cv.): ‘Oblica,’ the leading Croatian cultivar in orchards; ‘Leccino,’ a proven sensitive cultivar to salinity and drought (Perica et al., 2008; Tattini and Traversi, 2009; Rossi et al., 2016); and cultivar ‘Koroneiki,’ recognized for its resistance to unfavorable growing conditions (Chartzoulakis et al., 2002).

### 2.3 Experimental design and treatment application

Following a three-month acclimatization period, the experimental design was set according to the principle of a random block arrangement in three replicates per treatment i.e., nine plants per genotype (control, NaCl-induced salt stress, mannitol-induced drought stress). The experiment commenced by exposing the olive plants to 150 mM NaCl (non-iodized salt; Solana Pag d.d., Pag, Croatia) and 300 mM mannitol (powder; Roquette, Lestrem, France), which were added to the ½ HNS. To prevent osmotic shock, isosmotic concentrations of NaCl (−0.83 MPa) and mannitol (−0.82 MPa) were gradually reached over three days, with daily increments of 50 mM NaCl and 100 mM mannitol. The water potential was determined midday on a fully developed young leaf using “PMS 1000” (Model 1000 Pressure Chamber, PMS Instrument Company, Oregon, USA). Control plants were irrigated only with ½ HNS. To mitigate edge effects, three olive plants (cv. ‘Canino’) were strategically positioned at the ends of the rows, ensuring equal osmotic potential values for sodium chloride and mannitol concentrations. Percolate from each treatment was collected and analyzed daily. The experiment lasted three weeks (21 days).

### 2.4 Morphometric parameters and ionic composition of roots and shoot leaves

Morphometric assessments of all plants in the experiment were carried out at three different time points: at the start, on the 12th day, and at the end. The final morphometric measurements took place in the laboratory, where the plants were cleaned of inorganic substrates and divided into root, stem, and leaf sections. It is noteworthy that the measured shoot indicates the growth from the main vegetative bud of the current year, which initiated the growth cycle in March. To ensure uniform measurement, all other vegetative shoots were removed from the lateral buds at the beginning of the vegetative growth stage. The morphometric parameters presented in this study included analysis of the shoot length, leaf surface area, and dry weight of leaves (Tadić et al., 2021). Leaf surface area was measured after the removal of the leaves. The measurement was done on the first four fully developed and mature leaves from the top of the shoot. Surface measurements of leaves were performed using an Epson Perfection V700 Photo scanner and WinFOLIA software. Before analyzing the dry mass, the plant material was dried at 75°C for 48 hours. The dried root and leaves underwent grinding for the analysis of anionic and cationic ionic composition in root and shoot leaves. Ionic content determination involved weighing 0.1 g of ground samples, followed by ultrasonic bath treatment, centrifugation, and measurement of pH and electrical conductivity. Dionex LC 30 Chromatography Oven, Dionex CD 20 Conductivity Detector, and GP 50 Gradient Pump were utilized for ionic content determination. K^+^ leakage was measured by selecting a fully developed leaf from the shoot tip of each sample. A 5 mm diameter disk was cut from each leaf and placed in a glass vial. The vials were filled with 30 ml of re-distilled water and kept in a dark room for 24 hours. After this period, the first measurement of K^+^ leakage was conducted on all samples using a Sherwood 410 flame spectrometer (Sherwood Scientific Ltd, Cambridge, United Kingdom). The samples that remained in the vials were then autoclaved in a Presoclave II 80 (J.P. Selecta, Abrera, Spain) at 120°C and 103.1 MPa for 20 minutes. They were then left to cool in a dark room for another 24 hours before the second measurement of K^+^ leakage was performed. K^+^ leakage was calculated from the following expression: KL= KL1/(KL1+KL2) x 100.

### 2.5 Physiological and biochemical parameters

On the last day of the experiment, the first four fully developed leaves from the top of each shoot were collected, sealed in polyvinyl chloride bags, and quickly submerged in liquid nitrogen for several seconds. These samples were then lyophilized for five days using a Labconco FreeZone 2.5 lyophilizer (Labconco Corporation, Kansas City, MO, USA) and stored at -65°C until analysis. The leaves were homogenized for one minute using an IST400 mixer mill (InSolido Technologies, Zagreb, Croatia). For the analysis of antioxidative enzymes, powdered material (50 mg) was homogenized in potassium phosphate buffer (pH 7.0) for one more minute. The homogenates were centrifuged at 25,000 × g for 30 minutes at 4°C using a Sigma 3K18 centrifuge (Osterode am Harz, Germany), and the supernatants were examined for enzyme activity and soluble proteins according to Bradford (1976). The protein contents of the enzyme extracts were determined using bovine serum albumin (Sigma-Aldrich) as a standard and expressed as mg/g DW. The activity of SOD was determined by measuring its ability to inhibit the reduction of nitroblue tetrazolium (Sigma-Aldrich) by superoxide according to the method of Beauchamp and Fridovich (1971), with bovine SOD used for calibration. The activity of GPOX was measured using guaiacol as a substrate following the procedure outlined by Chance and Maehly (1955). The formation of tetraguaiacol was monitored at 470 nm and quantified using its extinction coefficient (26.6 mM/cm). The contents of malondialdehyde (MDA), an indicator of lipid peroxidation, and proline were determined according to Radić et al. (2009). The MDA content was measured using the thiobarbituric acid method at 532 nm and expressed as nmol/mg DW. The proline content was estimated using the ninhydrin reagent, with L-proline (Sigma-Aldrich) as a standard, and the absorbance was read at 520 nm. Proline content was expressed as mg/g DW.

### 2.6 Statistical analysis

Statistical data analysis was performed using the program STATISTICA 13.3 (TIBCO, Inc., USA). Each data point is the average of three replicates (n=3) ± standard deviations (SD). To allow adequate comparison of olive genotypes, all data were normalized, with controls assigned a value of 1. The normality of the data was tested by Shapiro-Wilk’s W test. Statistical significance between control and treated samples, as well as between the treated samples themselves, was calculated by the parametric test one-way analysis of variance (ANOVA) followed by Duncan’s New Multiple Range Test, i.e. *post hoc* test of multiple comparisons. Statistically significant differences at the significance level of 0.05 (p < 0.05) between control and treated plants are shown in different letters. The original data are presented in auxiliary tables in the appendices.

## 3 Results

### 3.1 Impact of drought and salinity stress on morphometric measurements

**Figure 1:**
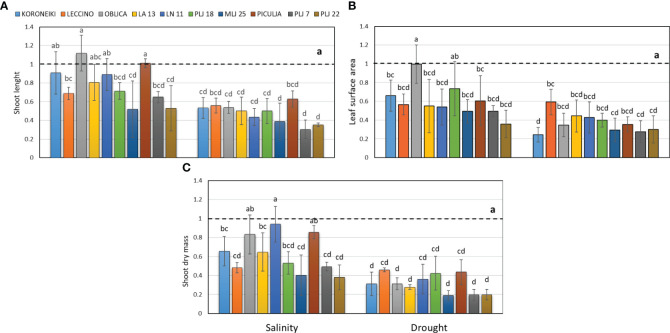
Shoot length **(A)**, leaf surface area **(B)** and shoot dry mass **(C)** in olive genotypes measured after exposure to 150 mM NaCl or 300 mM mannitol during 21 days of experiment. Controls are normalized to the value 1. Data are presented as means ± standard deviations, n=3. Bars not sharing any letter are significantly different by Duncan’s New Multiple Range Test at p ≤ 0.05. Raw data are presented in **Supplementary Table 1**.

In this study, wild olives (LA 13, LN 11, PLJ 18, MLJ 25, ‘Piculja’, PLJ 7, and PLJ 22) were analyzed and compared with reference cultivars ‘Koroneiki,’ ‘Oblica,’ and ‘Leccino’. The research revealed that drought stress had a more pronounced impact on the growth parameters of most cultivated and wild olives. Drought treatment led to a significant reduction of shoot length, leaf surface, and shoot dry mass in all olive genotypes (**Figure 1**). The salinity treatment had different effects on morphological characteristics (**Figure 1**) among the analyzed genotypes. The shoot length of salt-treated ‘Koroneiki’ and ‘Oblica’ cultivars was similar to the control, while cv. ‘Leccino’ exhibited a significant reduction in that growth parameter under salinity (**Figure 1A**). Among wild olives, genotypes PLJ 18, MLJ 25, PLJ 7 and PLJ 22 had significantly reduced shoot length in response to salinity treatment, while the parameter in ‘Piculja’ genotype did not significantly differ from control (**Figure 1A**). Unlike cv. ‘Oblica’, leaf surface area of cv. ‘Koroneiki’ and ‘Leccino’ significantly decreased under salinity treatment. Except PLJ 18, a significant reduction of leaf surface area was observed in wild olives under salinity treatment (**Figure 1B**). Shoot dry mass of most olive genotypes significantly decreased under salinity stress compared to control. That growth parameter was not significantly affected by salinity treatment only in ‘Oblica’, LN 11, and ‘Piculja’ (**Figure 1C**).

### 3.2 Impact of drought and salinity stress on sodium and chlorine content in olive leaves and roots

**Figure 2:**
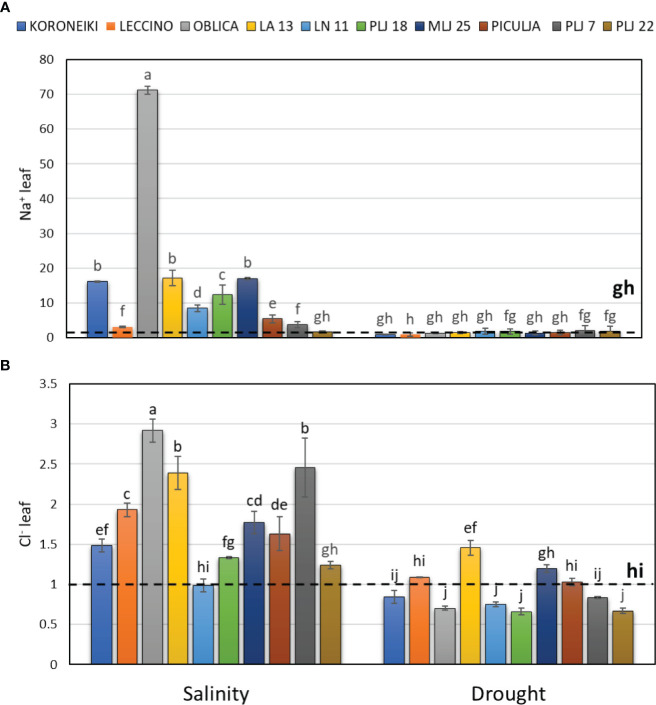
Relative content of leaf Na^+^ **(A)** and Cl^-^ **(B)** in olive genotypes measured after exposure to 150 mM NaCl or 300 mM mannitol during 21 days of experiment. Controls are normalized to the value 1. Data are presented as means ± standard deviations, n=3. Bars not sharing any letter are significantly different by Duncan’s New Multiple Range Test at p ≤ 0.05. Raw data are presented in **Supplementary Table 2**.

**Figure 3:**
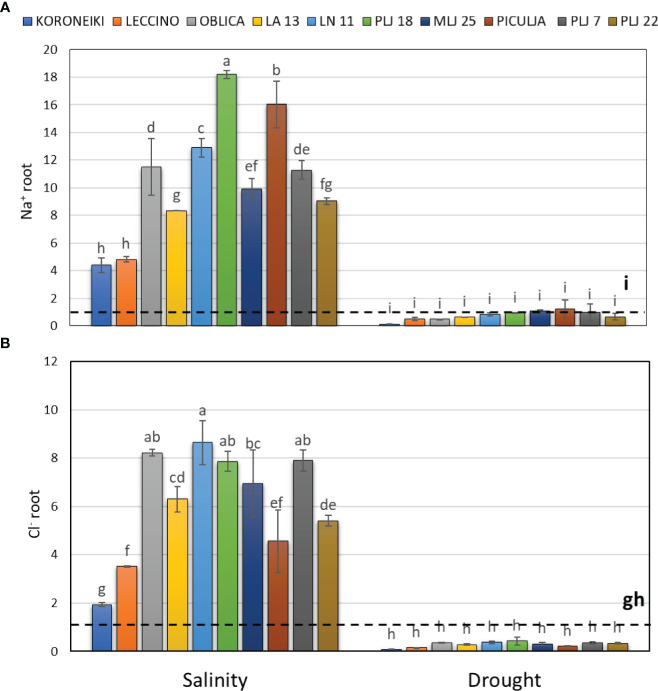
Relative content of root Na^+^ **(A)** and Cl^-^ **(B)** in olive genotypes measured after exposure to 150 mM NaCl or 300 mM mannitol during 21 days of experiment. Controls are normalized to the value 1. Data are presented as means ± standard deviations, n=3. Bars not sharing any letter are significantly different by Duncan’s New Multiple Range Test at p ≤ 0.05. Raw data are presented in **Supplementary Table 2**.

The salinity treatment significantly affected salt ion content, particularly Na^+^, in both shoot leaves (**Figure 2**) and roots (**Figure 3**). The highest accumulation of Na^+^ was noted in the leaves of reference cv. ‘Oblica’ (**Figure 2A**). Other olive genotypes also displayed a significant increase of Na^+^ values in leaves under salinity, except for genotype PLJ 22. Leaf Na^+^ content of LA 13, LN 11, PLJ 18, and MLJ 25 genotypes was comparable to that of cv. ‘Koroneiki’, while leaf Na^+^ content of ‘Piculja’, PLJ 7, and PLJ 22 was more similar to that of cv. ‘Leccino’. Regarding leaf Cl^-^ content, salinity treatment mostly increased levels of that anion in olive trees (**Figure 2**). The highest Cl^-^ content was measured in cv. ‘Oblica’ followed by LA13 and PLJ7 genotypes. Leaf Cl^-^ content of salt-treated LN 11 and PLJ 22 genotype was similar to the control (**Figure 2B**). Drought treatment did not affect leaf Na^+^ and Cl^-^ levels, except wild genotype LA 13 which showed a significant increase in Cl^-^ content (**Figure 2B**) compared to control.

Salinity treatment led to significant Na^+^ accumulation in roots of all olive genotypes (**Figure 3**), with wild genotype PLJ 18 having the highest increase of the ion followed by genotype ‘Piculja’. Wild olives had at least two times higher root Na^+^ content than reference cv. ‘Koroneiki’ and ‘Leccino’ (**Figure 3A**). Roots of all genotypes showed significantly increased Cl^-^ values under salinity except cv. ‘Koroneiki’ (**Figure 3B**). Drought treatment did not affect salt ions in roots.

### 3.3 Impact of drought and salinity stress on mineral ion content in olive leaves and roots

In general, stress treatments had opposite effects on leaf and root contents of measured macroelements (**Table 2**). Regardless of the treatment, Mg^2+^ and Ca^2+^ contents of roots were less reduced than leaf values of those ions, while root K^+^ content was more affected than leaf content of that element in most olive genotypes. Leaf Mg^2+^ content of almost all genotypes was significantly reduced under both treatments, except for genotype PLJ7 where the content of that macroelement was similar to control (**Table 2**). Another exception was recorded for wild genotype LA 13, which showed a significant increase in leaf Mg^+^ content under drought treatment. Reference cv. Koroneiki and Oblica and genotype ‘Piculja’ exhibited decreased Ca^2+^ content under both treatments, while the content of that macroelement increased in LN 11, MLJ 25, and PLJ 7 genotypes under drought treatment. Leaf K^+^ values were reduced in ‘Koroneiki’ in response to both treatments, in PLJ 7, PLJ 22, and ‘Piculja’ genotypes in response to drought and in ‘Oblica’ in response to salinity. Regarding K^+^ leakage, that parameter significantly increased only in cv. ‘Oblica’ and LN 11 under salinity and drought treatment, respectively (**Table 2**). Although other olive genotypes mostly displayed a rise in K^+^ leakage, it was not significant compared to the control treatment. Mg^+^ content in roots of olive genotypes (**Table 3**) generally showed no significant difference from the control treatment. Lower content of that element was detected in PLJ 18 under salinity, LN 11 under drought, and PLJ 7 genotype under both treatments. Values of Ca^2+^ significantly increased in ‘Koroneiki,’ ‘Leccino,’ as well as genotypes LA 13 and MLJ 25 under drought, and significantly decreased in genotypes LN 11 and PLJ 18 under salinity treatment (**Table 3**). Almost all genotypes experienced a decline in K^+^ values under both treatments except for cv. ‘Oblica’ in salinity treatment. Also, wild genotype PLJ 18 exhibited an increase in K^+^ content under drought conditions (**Table 3**).

**Table 2:** Relative ion content (Mg^2+^, Ca^2+^, K^+^) and K^+^ leakage in shoot leaves of reference cultivars and wild olive genotypes after exposure to 150 mM NaCl or 300 mM mannitol during 21 day.

Genotype	Treatment	Mg^2+^	Ca^2+^	K^+^	K^+^ leakage
**Koroneiki**	Control Salinity Drought	1.00 (0.029) ^b*^ 0.80 (0.010) **^c^** 0.62 (0.038) **^ef^**	1.00 (0.038) **^b^** 0.77 (0.033) **^cd^** 0.69 (0.009) **^cde^**	1.00 (0.029) **^a^** 0.71 (0.012) **^e^** 0.59 (0.010) **^f^**	1.00 (0.343) **^c^** 1.32 (0.422) **^bc^** 1.06 (0.439) **^bc^**
**Leccino**	Control Salinity Drought	1.00 (0.006) **^b^** 0.75 (0.088) **^de^** 0.62 (0.022) **^ef^**	1.00 (0.049) **^b^** 0.88 (0.084) **^bc^** 0.72 (0.090) **^cd^**	1.00 (0.090) **^a^** 0.95 (0.039) **^ab^** 0.93 (0.096) **^ab^**	1.00 (0.167) **^c^** 1.61 (0.205) **^bc^** 1.34 (0.382) **^bc^**
**Oblica**	Control Salinity Drought	1.00 (0.038) **^b^** 0.73 (0.081) **^de^** 0.55 (0.009) **^fg^**	1.00 (0.063) **^b^** 0.36 (0.016) **^ef^** 0.27 (0.011) **^f^**	1.00 (0.189) **^a^** 0.87 (0.038) **^bc^** 1.01 (0.059) **^a^**	1.00 (0.171) **^c^** 2.90 (0.674) **^a^** 1.72 (0.020) **^bc^**
**LA 13**	Control Salinity Drought	1.00 (0.021) **^b^** 0.55 (0.117) **^fg^** 1.64 (0.144) **^a^**	1.00 (0.057) **^b^** 0.54 (0.038) **^de^** 0.94 (0.049) **^bc^**	1.00 (0.007) **^a^** 0.95 (0.007) **^ab^** 0.99 (0.077) **^a^**	1.00 (0.503) **^c^** 1.30 (0.211) **^bc^** 1.75 (0.529) **^bc^**
**LN 11**	Control Salinity Drought	1.00 (0.034) **^b^** 0.76 (0.017) **^cd^** 0.73 (0.039) **^de^**	1.00 (0.138) **^b^** 1.08 (0.107) **^ab^** 1.33 (0.173) **^a^**	1.00 (0.010) **^a^** 0.94 (0.033) **^ab^** 0.86 (0.034) **^bcd^**	1.00 (0. 396) **^c^** 1.30 (0.185) **^bc^** 2.08 (1.024) **^ab^**
**PLJ 18**	Control Salinity Drought	1.00 (0.034) **^b^** 0.63 (0.044) **^ef^** 0.80 (0.056) **^cd^**	1.00 (0.138) **^b^** 0.80 (0.141) **^bcd^** 1.08 (0.248) **^ab^**	1.00 (0.010) **^a^** 0.99 (0.021) **^a^** 0.82 (0.014) **^cd^**	1.00 (0. 155) **^c^** 1.71 (0.529) **^bc^** 1.53 (0.411) **^bc^**
**MLJ 25**	Control Salinity Drought	1.00 (0.034) **^b^** 0.57 (0.031) **^fg^** 0.53 (0.086) **^g^**	1.00 (0.138) **^b^** 0.99 (0.100) **^b^** 1.22 (0.159) **^a^**	1.00 (0.010) **^a^** 0.92 (0.026) **^ab^** 0.85 (0.073) **^bcd^**	1.00 (0. 581) **^c^** 1.16 (0.134) **^bc^** 1.15 (0.325) **^bc^**
**Piculja**	Control Salinity Drought	1.00 (0.021) **^b^** 0.77 (0.051) **^cd^** 0.87 (0.023) **^bc^**	1.00 (0.167) **^b^** 0.58 (0.061) **^de^** 0.58 (0.010) **^de^**	1.00 (0.046) **^a^** 0.96 (0.021) **^ab^** 0.86 (0.023) **^bcd^**	1.00 (0.204) **^c^** 1.18 (0.718) **^bc^** 0.95 (0.790) **^c^**
**PLJ 7**	Control Salinity Drought	1.00 (0.034) **^b^** 0.93 (0.083) **^bc^** 0.93 (0.069) **^b^**	1.00 (0.030) **^b^** 0.91 (0.125) **^bc^** 1.24 (0.173) **^a^**	1.00 (0.029) **^a^** 0.91 (0.089) **^bc^** 0.77 (0.029) **^de^**	1.00 (0. 153) **^c^** 1.51 (1.024) **^bc^** 1.02 (0.148) **^c^**
**PLJ 22**	Control Salinity Drought	1.00 (0.034) **^b^** 0.92 (0.124) **^bc^** 0.67 (0.123) **^def^**	1.00 (0.077) **^b^** 0.99 (0.133) **^b^** 0.99 (0.135) **^b^**	1.00 (0.012) **^a^** 1.00 (0.069) **^a^** 0.79 (0.019) **^d^**	1.00 (0. 308) **^c^** 1.15 (0.868) **^bc^** 1.12 (0.751) **^bc^**

*Controls are normalized to the value 1. Data are presented as means ± SD (parenthesis), n=3. SD values in the control treatment were calculated relative to the SD values of the raw data for control. Means not sharing any letter within column are significantly different by Duncan’s New Multiple Range Test at p ≤ 0.05. Raw data are presented in **Supplementary Table 3**.

**Table 3:** Relative ion content (Mg^2+^, Ca^2+^, K^+^) in roots of reference cultivars and wild olive genotypes after exposure to 150 mM NaCl or 300 mM mannitol during 21 day experiment.

Genotype	Treatment	Mg^2+^	Ca^2+^	K^+^
**Koroneiki**	Control Salinity Drought	1.00 (0.037) ^ab*^ 0.99 (0.110) **^ab^** 1.22 (0.061) **^a^**	1.00 (0.085) **^def^** 0.84 (0.051) **^efg^** 1.85 (0.117) **^b^**	1.00 (0.042) **^b^** 0.84 (0.012) **^c^** 0.58 (0.010) **^def^**
**Leccino**	Control Salinity Drought	1.00 (0.089) **^ab^** 0.97 (0.218) **^abc^** 0.92 (0.037) **^bc^**	1.00 (0.020) **^def^** 0.97 (0.208) **^def^** 1.53 (0.102) **^c^**	1.00 (0.037) **^b^** 0.48 (0.009) **^fg^** 0.50 (0.158) **^fg^**
**Oblica**	Control Salinity Drought	1.00 (0.113) **^ab^** 0.95 (0.162) **^bc^** 0.93 (0.009) **^bc^**	1.00 (0.168) **^def^** 0.89 (0.223) **^efg^** 1.28 (0.089) **^cd^**	1.00 (0.011) **^b^** 0.90 (0.041) **^bc^** 0.66 (0.039) **^de^**
**LA 13**	Control Salinity Drought	1.00 (0.237) **^ab^** 1.06 (0.088) **^ab^** 1.03 (0.119) **^ab^**	1.00 (0.033) **^def^** 1.04 (0.152) **^def^** 1.52 (0.504) **^c^**	1.00 (0.130) **^b^** 0.71 (0.066) **^d^** 0.50 (0.031) **^fg^**
**LN 11**	Control Salinity Drought	1.00 (0.221) **^ab^** 0.85 (0.348) **^bcd^** 0.76 (0.204) **^cd^**	1.00 (0.527) **^def^** 0.55 (0.280) **^gh^** 1.13 (0.184) **^de^**	1.00 (0.089) **^b^** 0.79 (0.089) **^cd^** 0.35 (0.056) **^h^**
**PLJ 18**	Control Salinity Drought	1.00 (0.013) **^ab^** 0.71 (0.095) **^cd^** 1.17 (0.221) **^a^**	1.00 (0.059) **^def^** 0.44 (0.091) **^h^** 1.12 (0.176) **^de^**	1.00 (0.018) **^b^** 0.56 (0.032) **^ef^** 1.19 (0.219) **^a^**
**MLJ 25**	Control Salinity Drought	1.00 (0.042) **^ab^** 0.95 (0.039) **^bc^** 1.01 (0.021) **^ab^**	1.00 (0.048) **^def^** 0.77 (0.068) **^fg^** 2.16 (0.311) **^a^**	1.00 (0.019) **^b^** 0.63 (0.021) **^def^** 0.44 (0.026) **^gh^**
**Piculja**	Control Salinity Drought	1.00 (0.047) **^ab^** 0.97 (0.066) **^ab^** 1.12 (0.053) **^ab^**	1.00 (0.057) **^def^** 0.85 (0.027) **^efg^** 1.18 (0.041) **^de^**	1.00 (0.049) **^b^** 0.67 (0.008) **^de^** 0.47 (0.027) **^gh^**
**PLJ 7**	Control Salinity Drought	1.00 (0.045) **^ab^** 0.59 (0.055) **^d^** 0.67 (0.122) **^d^**	1.00 (0.021) **^def^** 0.70 (0.067) **^fgh^** 0.87 (0.047) **^efg^**	1.00 (0.043) **^b^** 0.48 (0.037) **^fg^** 0.61 (0.046) **^def^**
**PLJ 22**	Control Salinity Drought	1.00 (0.079) **^ab^** 0.85 (0.067) **^bcd^** 0.89 (0.055) **^bcd^**	1.00 (0.110) **^def^** 0.85 (0.071) **^efg^** 1.15 (0.047) **^de^**	1.00 (0.074) **^b^** 0.57 (0.069) **^def^** 0.44 (0.019) **^gh^**

*Controls are normalized to the value 1. Data are presented as means ± SD (parenthesis), n=3. SD values in the control treatment were calculated relative to the SD values of the raw data for control. Means not sharing any letter within column are significantly different by Duncan’s New Multiple Range Test at p ≤ 0.05. Raw data are presented in **Supplementary Table 4**.

### 3.4 Biochemical responses of olive genotypes to drought and salinity stress

**Figure 4:**
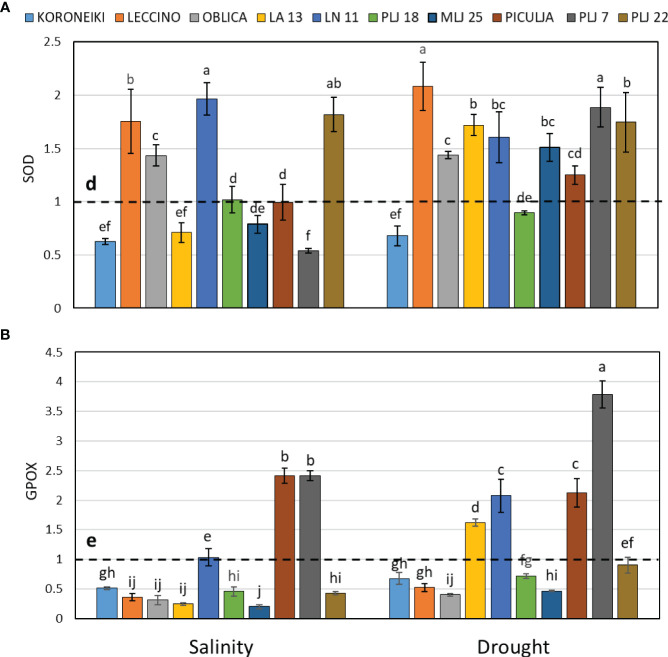
Relative activity of SOD **(A)** and GPOX **(B)** in olive leaves after exposure to 150 mM NaCl or 300 mM mannitol during 21 days of experiment. Controls are normalized to the value 1. Data are presented as means ± standard deviations, n=4. Bars not sharing any letter are significantly different by Duncan’s New Multiple Range Test at p ≤ 0.05. Raw data are presented in **Supplementary Table 5**.

**Figure 5:**
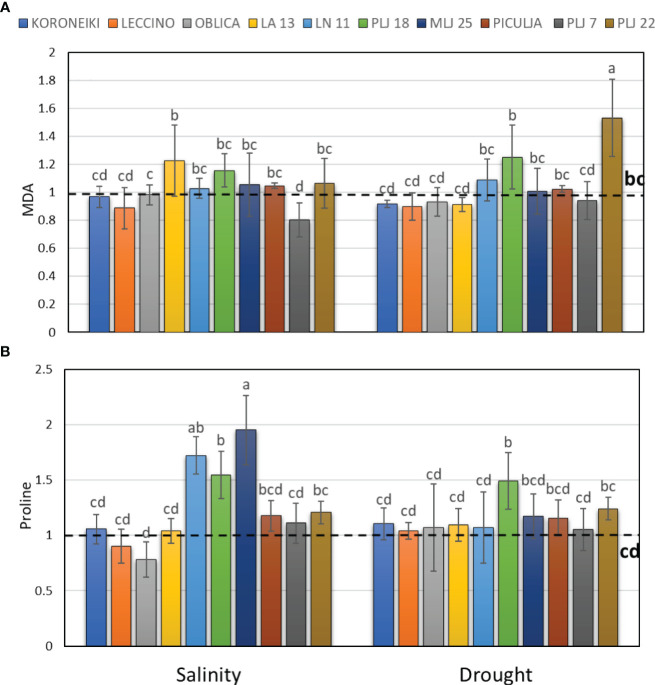
Relative content of malondialdehyde (MDA) **(A)** and proline **(B)** in olive leaves after exposure to 150 mM NaCl or 300 mM mannitol during 21 days of experiment. Controls are normalized to the value 1. Data are presented as means ± standard deviations, n=4. Bars not sharing any letter are significantly different by Duncan’s New Multiple Range Test at p ≤ 0.05. Raw data are presented in **Supplementary Table 5**.

Induction of guaiacol peroxidase was recorded in wild ‘Piculja’ and PLJ 7 genotypes under both treatments, (**Figure 4A**) and in wild LA 13 and LN 11 genotypes under drought. However, the activity of GPOX was significantly inhibited by stress in most olive genotypes including the reference cultivars (**Figure 4A**). Generally, drought caused greater stimulation of SOD activity than salinity in most olive genotypes (**Figure 4B**). Both stress conditions significantly increased SOD activity in cultivars ‘Leccino’ and ‘Oblica’, as well as in LN 11 and PLJ 22 genotypes. The activity of that antioxidative enzyme was inhibited in cv. ‘Koroneiki’ under both stressors and LA 13 and PLJ 7 genotypes under salinity. MDA content exhibited minimal changes overall in response to stress. Only genotype PLJ 22 (**Figure 5A**) showed a substantial increase in MDA content under drought stress (**Figure 5**). Salinity and drought stress had a weaker impact on proline content in reference cultivars (**Figure 5B**), while a significant increase was observed in LA 13, LN 11, and PLJ 18 genotypes (**Figure 5B**). Only genotype PLJ 18 showed considerable accumulation of proline under drought stress.

## 4 Discussion

### 4.1 Impact of drought and salinity stress on morphometric parameters

In this study, drought stress inhibited the growth of olive shoots and leaf area to a significant extent compared to the stress induced by increased salinity with NaCl. Cultivars ‘Koroneiki’ and ‘Oblica’ were expected to have reduced growth, but the values of shoot length were not significantly different compared to the control treatment. Similar results were reported by Bashir et al. (2021), who found a reduction of shoot growth in the tolerant cv. ‘Canino’ and the sensitive cv. ‘Sirole’ even at lower NaCl concentrations (50 mM). Similar results were recorded by Perica et al. (2008), where the effect of salinity was linear, and there was a quadratic reduction in shoot length and dry mass in olive cultivars. Olive leaves with densely packed mesophyll limited the entry of CO_2_ reducing the biomass synthesis (Bongi and Loreto, 1989; Tattini et al., 1995; Chartzoulakis et al., 2002). The difference in the effects of NaCl and mannitol on olive growth is likely associated with different osmotic adaptations. Using Na^+^ and Cl^-^ for osmotic adjustment is less energetically demanding and more cost-effective than the biosynthesis of organic solutes, as long as the salt ions are sequestered in cell vacuoles (Munns et al., 2020).

### 4.2 Impact of drought and salinity stress on sodium and chlorine content in olive leaves and roots

The effectiveness of *Olea europaea* L. in protecting sensitive shoots from the harmful effects of high concentrations of toxic ions may be due to its moderate growth dynamics and reduced water transport from the root zone with elevated NaCl concentration, known as the “low sodium strategy” (Chartzoulakis, 2005; Melgar et al., 2006). In this study, the accumulation of harmful Na^+^ and Cl^-^ ions was observed in the olive leaves and roots in significantly higher levels under salinity treatment, while the content of salt ions did not differ from the control under drought treatment. The roots of the ‘Koroneiki’ and ‘Leccino’ cultivars displayed lower Na^+^ values than those of wild olives, though notably higher than those in the control treatment. Although the ‘Oblica’ cultivar utilizes the same resistance mechanism (Melgar et al., 2009) to salinity, the translocation of Na^+^ from roots to shoot leaves was several times higher than in other genotypes. As the cultivar ‘Oblica’ showed the highest shoot length among all genotypes (**Figure 2A**), it can be assumed that the distribution of Na^+^ ions into the vacuoles of leaf and root cells was relatively efficient and maintained osmotic pressure in the cells. Contrary to all previous studies (Perica et al., 2008; Tattini and Traversi, 2009; Rossi et al., 2016), in this study ‘Leccino’ cultivar showed low or control-treatment-like leaf Na^+^ values, depending on the observed group. In the aforementioned research, the highest amount of Na^+^ was usually recorded in the leaves of the ‘Leccino’ compared to other cultivars. By comparing the results of this study with short-term salinity exposure to studies with long-term salinity exposure (Goreta et al., 2007; Perica et al., 2008; Melgar et al., 2009), we assume that the exposure time and salinity intensity is the key to different dynamics of salt ion uptake, in these cases Na^+^ and Cl^-^. Studies show that compared to Na^+^, olive trees are less sensitive to Cl^−^, which generally is not phytotoxic to olive trees. The Cl^−^ likely contributed to osmotic adjustment, although to a lesser extent than Na^+^ (Melgar et al., 2009). This would explain the early perception of stress in more sensitive cultivars like ‘Leccino’, which responded quickly by reducing shoot growth intensity (**Figure 2A**) and preventing the translocation of harmful Na^+^ ions to sensitive shoot leaves (**Figure 3A**). Storey and Walker (1998) concluded that certain citrus genotypes sensitive to high salinity require a longer time to accumulate Na^+^ and Cl^-^ ions to a stable level than salinity-tolerant genotypes. This highlights the varying responses of olive ion content to drought and salinity stress across different genotypes.

Developed root system, also plays a significant role in the resistance to abiotic stresses such as salinity and drought (Rossi et al., 2016). In our study, both treatments negatively affected leaf surface area, shoot length, and dry mass of the ‘Leccino’ cultivar. The recorded results are consistent with the research of Perica et al. (2008) where the ‘Leccino’ showed the greatest growth reduction compared to other cultivars. Earlier studies on root morphology and natural branching suggest tight control of potentially toxic ion uptake (Tattini et al., 1997). The latter authors hypothesized that longer root length allows the plant to reach deeper soil layers with lower localized NaCl concentration compared to shallow soil layers, where, for example, the ‘Leccino’ cultivar roots. To avoid such a genotype-specific trait that would certainly impact results in a relatively small volume of growth containers, in this study the plants were grown in a highly permeable inorganic substrate with a small mass-to-volume ratio and were adapted over a relatively long period to ensure well-developed and evenly distributed roots.

### 4.3 Impact of drought and salinity stress on mineral ion content in olive leaves and roots

Osmotic adaptation in olive shoot leaves under abiotic stress conditions like salinity and drought could also be achieved by the accumulation of K^+^ and Ca^2+^ (Tattini and Traversi, 2009). This was confirmed by the results for the ion content in olive shoot leaves, particularly for representatives of sensitive olives, such as the ‘Leccino’ cultivar. The results obtained showed that the cv.’Leccino’ was able to maintain a sufficient concentration of K^+^ in the cytoplasm of shoot leaf cells under both stress conditions. Tattini and Traversi (2009) reported that olives supplemented with Ca^2+^ under high salinity treatment showed a better ability to translocate Na^+^ to the plant shoots. This is consistent with the results obtained for most salt-treated wild olive genotypes, as these genotypes achieved Ca^2+^ values similar to the control treatment.

Potassium leakage from plant cells is often used as an indicator of tissue damage caused by stress, which includes K^+^ leakage and certain counterions (Demidchik et al., 2014). Research suggests the involvement of reactive oxygen species (ROS), particularly hydroxyl radicals and hydrogen peroxide, in the activation of protein channels for K^+^ efflux from the cell (Demidchik et al., 2014). Thus, increased values of K^+^ leakage recorded in a few olive genotypes could be related to higher amounts of ROS generated by the action of NADPH oxidase or another source (Demidchik et al., 2014). The results obtained imply that the limitation of K^+^ uptake from the soil, caused by the osmotic component of stress (Wang et al., 2013), was halted, and the ion stress phase induced by Na^+^ and Cl^-^ ions began. It was assumed that proton pumps in the root cells of more sensitive cultivars function at a significantly lower level compared to those in more resistant cultivars, enabling the latter to uptake significantly larger amounts of K^+^ under high salinity conditions (Maathuis and Amtmann, 1999). Also, Chartzoulakis (2005) suggested that the preferential accumulation of toxic ions in older leaves represents one of the mechanisms for preventing excessive salt accumulation in young shoot leaves. In the latter studies, the lowest K^+^ content was seen in the roots and older leaves. This implies that olives can keep high levels of K^+^ in young leaves, which can serve as the primary osmotic regulator for monovalent cations when encountering higher salinity levels. The reduction in K^+^ ions in the olive roots, resulting in a low K^+^/Na^+^ ratio, may indicate a mechanism by which the olive plant achieves ionic balance after receiving high concentrations of Na^+^ in the roots (Maathuis and Amtmann, 1999).

### 4.4 Biochemical responses of olive genotypes to drought and salinity stress

In the study by Goreta et al. (2007), the ‘Leccino’ cultivar exposed to gradually increasing salinity showed an initial increase in SOD enzyme activity, which later decreased with prolonged exposure (90 days) to high salinity. Conversely, the more salt-resistant cv. ‘Oblica’ exhibited an inverse pattern of SOD activity change, suggesting a more efficient adaptation mechanism to high salinity compared to ‘Leccino’. The results of that study are not following our results as both cultivars ‘Oblica’ and ‘Leccino’ displayed elevated SOD activities after 21 days of exposure. The reason for the discrepancy between the two studies could be due to different experimental setups such as duration of exposure and different SOD assay protocols. Namely, other signs of stress may emerge as the experiment progresses, leading to a reduction in SOD enzyme activity as H_2_O_2_ is further degraded by catalase and peroxidases (Ben-Ahmed et al., 2006). In this study, guaiacol peroxidase (GPOX) activity was affected by stress, mostly resulting in inhibition. Those results together with the observed induction of SOD activity and absence of lipid peroxidation in most olive genotypes, infer that some other enzyme, such as ascorbate peroxidase or catalase has a main role in the degradation of H_2_O_2_. Del Buono et al. (2021) also recorded the reduced activity of this enzyme in the cultivar ‘Arbequina’ exposed to the same salt concentration used in this study but for a longer period (40 days).

MDA content exhibited minimal changes overall in response to stress. Contrary to the findings of this study, high salinity and drought caused an increase in lipid peroxidation in the ‘Chétoui’ cultivar after 21 days under both conditions (Ben Abdallah et al., 2018). It is worth noting that the referenced study employed a higher NaCl concentration (200 mM) and imposed drought stress by employing reduced irrigation. However, by reducing irrigation throughout the experiment, the osmotic potential cannot be controlled or maintained at the same level, which likely contributed to a higher level of oxidative stress.

Besides acting as an excellent osmoprotectant, proline is believed to have three main roles during stress - it may act as a metal chelator, as an antioxidant, and as a signaling molecule (Ayaz et al., 2021). In our study, salinity and drought stress did not significantly impact the proline levels in the reference cv. and wild olive genotypes ‘Piculja’, PLJ7, and PLJ22. However, a significant increase was observed in the olive genotypes LN11, PLJ18, and MLJ25. Although proline increased in some wild genotypes, the recorded levels of this amino acid in shoot leaves of most tested olive genotypes do not support its role as the main osmolyte in adaptation to salinity and drought. Such results are in line with the findings of some authors (Bashir et al., 2021) who also determined variability in the proline levels and ascribed it to the difference in olive genotype, treatment intensity, and duration. Failure to track the trend of proline content increase/decrease could lead to misleading conclusions about plant stress levels. Despite its role as a signaling molecule in indicating changes in reactive ROS concentration, proline content might not reliably indicate stress levels, as suggested by this research and previous studies.

## 5 Conclusions

In this study, shoot length, dry mass, and leaf area were identified as significant indicators of drought stress levels in the analyzed genotypes. The presence of Na^+^ and Cl^-^ in the leaves of ‘Oblica’ cultivar presumably maintains osmotic pressure in cells since, despite their high values, other results did not indicate significant signs of stress. The most efficient energy response of olive trees to salinity and drought in a short period appears to be the uptake of Ca^2+^. The trend of changes in SOD enzyme activity suggests that it can serve as an indicator of salinity and drought. Different genotypes activate different defense mechanisms, ranging from the accumulation of inorganic ions to antioxidant enzymes and osmolyte synthesis, starting with the least energy-demanding ones. However, based on the results, it is crucial to recognize that wild olive genotypes do not inherently ensure superior plant traits. While wild plant relatives may offer genetic diversity and potential traits beneficial for breeding programs, such as resistance to pests or diseases, the transfer of desirable traits to cultivated plants isn’t guaranteed.

Earlier sampling of plant tissue could provide more concrete results of biochemical parameters, allowing for a more precise determination of stress levels compared to morphometric measurements. Two genotypes from the group of wild olives, LA 13 and PLJ 18, with their satisfactory results in morphometric parameters and biochemical analysis, deserve further research based on genotypic and physiological characterization, such as fruit size, and oil quality assessment. Understanding the diverse responses of olive genotypes to these stressors can aid in developing strategies for stress-resistant olive cultivars and improving agricultural practices in regions prone to salinity and drought.

## Data availability statement

The datasets presented in this study can be found in online repositories. The names of the repository/repositories and accession number(s) can be found in the article/**Supplementary Material**.

## Author contributions

JT: Conceptualization, Formal analysis, Funding acquisition, Investigation, Methodology, Resources, Visualization, Writing – original draft, Writing – review & editing. GD: Conceptualization, Resources, Writing – review & editing. MVB: Formal analysis, Writing – review & editing. SV: Formal analysis, Writing – review & editing. ZL: Formal analysis, Funding acquisition, Supervision, Writing – review & editing. SRB: Formal analysis, Methodology, Resources, Supervision, Visualization, Writing – original draft, Writing – review & editing.
