# Supplementary material for: Comparative analysis of cultivated and wild olive genotypes to salinity and drought stress
Source: Front Plant Sci. 2024 Jul 16;15:1423761. doi: 10.3389/fpls.2024.1423761 (PMC11286399; doi:10.3389/fpls.2024.1423761)
Supplement: Supplementary file 1 [file DataSheet_1.pdf]

Supplementary Material

Comparative analysis of cultivated and wild olive genotypes to salinity and drought stress

Josip Tadić [1,2], Gvozden Dumičić [1], Maja Veršić Bratinčević [3], Sandra Vitko [4], Zlatko Liber [2,4], Sandra Radić Brkanac[4*]

[1]Department of Plant Sciences, Institute for Adriatic Crops and Karst Reclamation, Split, Croatia

[2]Centre of Excellence for Biodiversity and Molecular Plant Breeding (CoE CroPBioDiv), Zagreb, Croatia

[3]Department of Applied Sciences, Institute for Adriatic Crops and Karst Reclamation, Split, Croatia

[4]Division of Botany, Department of Biology, Faculty of Science, University of Zagreb, Zagreb, Croatia

* Correspondence:

Corresponding Author

sandra.radic.brkanac@biol.pmf.hr

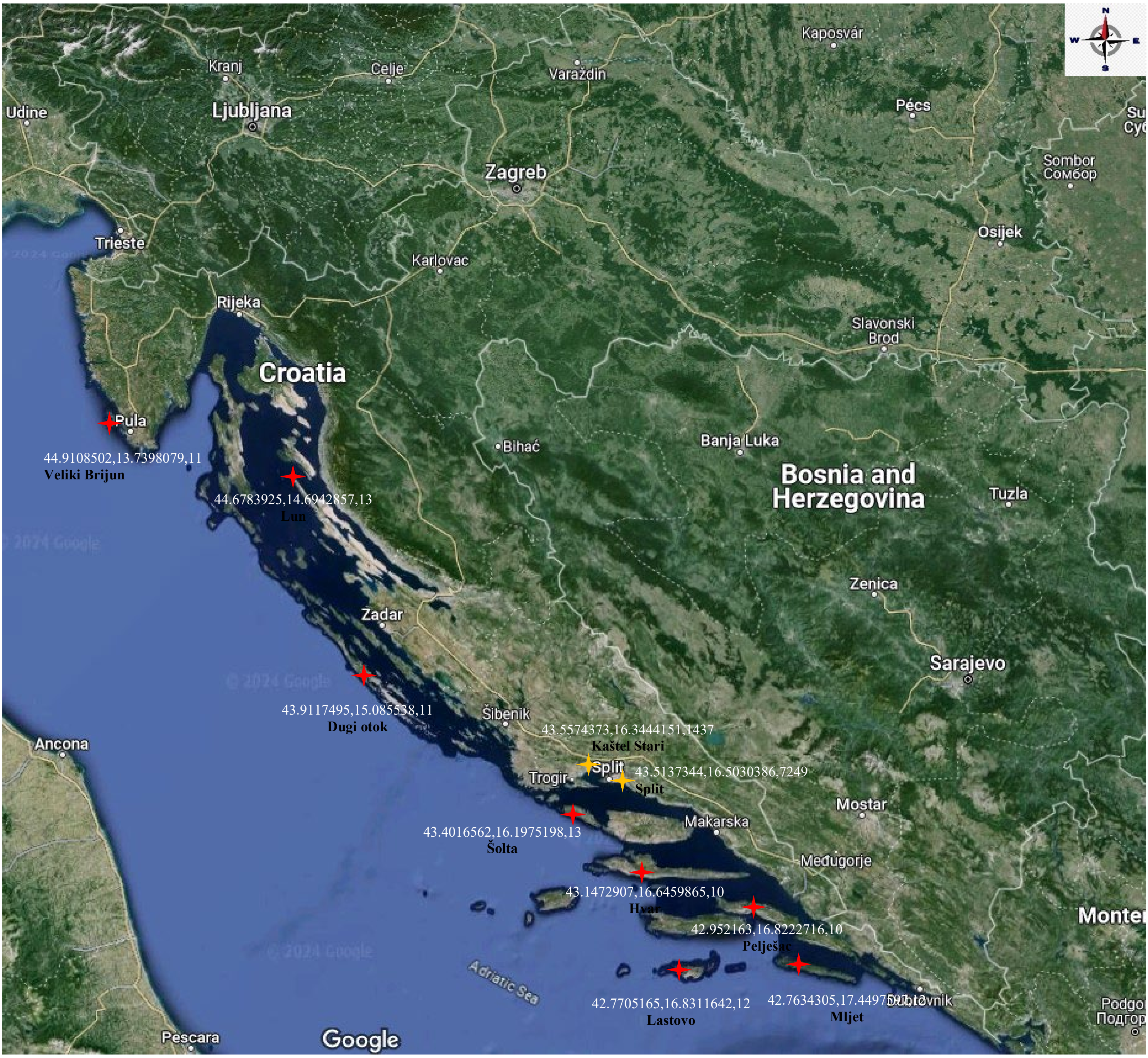

Supplementary Figure 1: Locations of areas from which olive genotypes were collected. Red stars (✦) on map are locations of wild olive genotypes, orange stars (✦) are field collection of Institute for Adriatic Crops and Karst reclamation in Split and Kaštel Stari.

Supplementary Table 1: Morphometric Measurements

GENOTYPE	TREATMENT	Shoot length (cm)	Leaf surface area (cm^2)	Shoot dry mass (g)
Koroneiki	Control	31.6 (11.53)*	68.13 (42.68)	1.17 (0.320)
	Salinity	28.6 (7.11)	44.89 (11.30)	0.77 (0.299)
	Drought	16.8 (3.54)	16.51 (5.30)	0.36 (0.144)
Leccino	Control	18.2 (2.83)	44.57 (17.03)	0.28 (0.058)
	Salinity	12.5 (1.25)	25.23 (4.92)	0.13 (0.015)
	Drought	10.2 (1.45)	26.41 (6.06)	0.13 (0.006)
Oblica	Control	18.63 (10.17)	59.08 (34.62)	0.42 (0.40)
	Salinity	20.83 (3.56)	58.81 (12.08)	0.35 (0.09)
	Drought	10.03 (1.21)	20.52 (7.40)	0.13 (0.03)
LA 13	Control	25.40 (1.21)	22.72 (3.12)	0.40 (0.04)
	Salinity	20.47 (4.91)	12.50 (8.73)	0.26 (0.12)
	Drought	12.70 (3.73)	10.07 (3.86)	0.11 (0.01)
LN 11	Control	21.03 (0.72)	28.20 (0.86)	0.28 (0.02)
	Salinity	18.73 (3.60)	15.21 (5.42)	0.26 (0.08)
	Drought	9.17 (1.91)	12.05 (4.72)	0.10 (0.04)
PLJ 18	Control	28.00 (1.73)	28.02 (7.73)	0.53 (0.25)
	Salinity	20.00 (2.54)	20.58 (8.08)	0.28 (0.06)
	Drought	14.00 (3.80)	11.15 (2.08)	0.22 (0.15)
MLJ 25	Control	27.10 (1.82)	76.99 (10.54)	0.58 (0.14)
	Salinity	14.10 (8.21)	38.07 (9.44)	0.23 (0.12)
	Drought	10.50 (5.31)	22.60 (9.66)	0.11 (0.03)
Piculja	Control	23 (9.10)	46.0 (24.57)	0.46 (0.29)
	Salinity	23.23 (1.10)	27.80 (12.26)	0.39 (0.03)
	Drought	14.47 (1.98)	16.30 (3.70)	0.20 (0.06)
PLJ 7	Control	34.73 (3.40)	72.39 (23.15)	0.78 (0.32)
	Salinity	22.53 (2.05)	35.91 (4.26)	0.39 (0.04)
	Drought	10.57 (3.42)	19.94 (8.33)	0.15 (0.05)
PLJ 22	Control	33.83 (4.08)	43.53 (17.41)	0.73 (0.19)
	Salinity	17.93 (8.16)	15.56 (10.75)	0.28 (0.024)
	Drought	11.97 (0.68)	13.08 (6.35)	0.14 (0.04)

Data are presented as averages (SD in parenthesis), n=3

Supplementary Table 2: Sodium and Chlorine Content in Olive Leaves and Roots

GENOTYPE	TREATMENT	Na^+ (mg/L) leaf	Na^+ (mg/L) root	Cl^- (mg/L) leaf	Cl^- (mg/L) root
Koroneiki	Control	0.52 (0.02)*	20.56 (0.90)	7.69 (0.83)	57.47 (3.81)
	Salinity	8.41 (0.13)	90.58 (10.83)	11.41 (0.61)	111.35 (4.61)
	Drought	0.49 (0.03)	2.60 (0.14)	6.47 (0.63)	4.68 (0.10)
Leccino	Control	0.46 (0.15)	14.60 (0.37)	4.70 (0.31)	33.07 (3.03)
	Salinity	1.42 (0.07)	70.32 (2.81)	9.06 (0.40)	116.13 (1.17)
	Drought	0.39 (0.25)	7.49 (1.68)	5.12 (0.02)	4.98 (0.27)
Oblica	Control	0.31 (0.08)	5.93 (1.44)	9.62 (1.86)	14.10 (6.01)
	Salinity	21.95 (0.38)	68.20 (12.22)	28.08 (1.38)	115.98 (1.92)
	Drought	0.38 (0.04)	2.92 (0.07)	6.78 (0.23)	5.08 (0.06)
LA 13	Control	0.67 (0.31)	10.53 (2.18)	7.57 (0.08)	20.68 (2.06)
	Salinity	11.52 (1.52)	87.85 (5.00)	18.09 (1.55)	130.24 (10.95)
	Drought	1.01 (0.20)	6.69 (1.36)	11.04 (0.72)	5.78 (0.60)
LN 11	Control	0.91 (0.11)	5.06 (1.09)	7.63 (1.38)	10.63 (1.26)
	Salinity	7.69 (0.86)	65.19 (3.45)	7.53 (0.59)	91.90 (9.54)
	Drought	1.61 (0.76)	4.21 (0.66)	5.72 (0.26)	4.00 (0.55)
PLJ 18	Control	0.39 (0.09)	6.44 (0.15)	8.08(0.18)	8.94 (0.16)
	Salinity	4.79 (1.08)	63.71 (4.75)	7.53 (0.59)	70.26 (3.69)
	Drought	0.68 (0.65)	6.87 (0.44)	5.72 (0.26)	3.86 (1.47)
MLJ 25	Control	0.72 (0.17)	5.29 (0.23)	5.20 (0.19)	14.38 (2.76)
	Salinity	12.25 (0.18)	96.23 (1.42)	9.24 (0.72)	100.00 (19.71)
	Drought	0.92 (0.42)	4.85 (1.60)	6.23 (0.23)	4.48 (0.80)
Piculja	Control	0.35 (0.05)	4.98 (0.85)	5.58 (0.06)	11.53 (0.12)
	Salinity	1.87 (0.39)	79.92 (8.42)	9.11 (1.17)	69.26 (18.49)
	Drought	0.49 (0.21)	6.16 (3.16)	5.76 (0.26)	2.49 (0.10)
PLJ 7	Control	0.31 (0.12)	7.39 (0.31)	4.64 (0.30)	14.72 (2.56)
	Salinity	1.15 (0.29)	83.23 (4.97)	11.41 (1.71)	116.38 (6.48)
	Drought	0.59 (0.43)	7.27 (4.33)	3.88 (0.05)	5.34 (0.68)
PLJ 22	Control	0.83 (0.45)	8.03 (0.85)	10.71 (0.23)	18.49 (1.30)
	Salinity	1.28 (0.33)	72.47 (1.82)	13.28 (0.48)	99.98 (4.19)
	Drought	1.44 (1.27)	5.35 (2.00)	7.15 (0.36)	6.27 (0.43)

Data are presented as averages (SD in parenthesis), n=3

Supplementary Table 3: Mineral Ion Content in Olive Leaves

GENOTYPE	TREATMENT	Mg^{+} (mg/L)	Ca^{+} (mg/L)	K^{+} (mg/L)	K^{+} leakage (mg/L)
Koroneiki	Control	1.22 (0.04)*	4.28 (0.16)	82.88 (2.48)	6.93 (2.38)
	Salinity	0.98 (0.01)	3.32 (0.14)	59.07 (1.00)	9.16 (2.93)
	Drought	0.76 (0.05)	2.96 (0.04)	49.10 (0.86)	7.31 (3.04)
Leccino	Control	1.69 (0.01)	4.56 (0.22)	63.51 (5.77)	8.49 (1.42)
	Salinity	1.27 (0.15)	4.02 (0.38)	60.13 (2.51)	13.63 (1.74)
	Drought	1.05 (0.04)	3.30 (0.41)	58.97 (6.11)	11.39 (3.25)
Oblica	Control	1.60 (0.05)	4.20 (0.07)	67.44 (3.27)	5.01 (0.85)
	Salinity	1.16 (0.13)	3.51 (0.19)	58.66 (2.55)	14.54 (3.37)
	Drought	0.88 (0.01)	2.62 (0.11)	68.20 (3.95)	8.63 (0.10)
LA 13	Control	4.47 (0.25)	4.20 (0.40)	78.76 (2.28)	5.72 (2.88)
	Salinity	2.47 (0.52)	2.26 (0.16)	75.00 (0.57)	7.43 (1.21)
	Drought	7.31 (0.65)	3.94 (0.20)	78.51 (6.03)	9.99 (3.02)
LN 11	Control	2.87 (0.79)	2.61 (1.05)	69.73 (5.31)	4.59 (1.82)
	Salinity	2.19 (0.05)	2.83 (0.28)	65.77 (2.27)	6.00 (1.02)
	Drought	2.10 (0.11)	3.48 (0.45)	59.75 (2.36)	9.56 (4.70)
PLJ 18	Control	2.79 (0.61)	3.37 (0.72)	71.02 (1.42)	5.83 (0.90)
	Salinity	1.74 (0.12)	2.71 (0.48)	70.68 (1.46	9.98 (3.08)
	Drought	2.22 (0.16)	3.65 (0.84)	58.36 (1.01)	8.89 (2.40)
MLJ 25	Control	3.51 (0.60)	5.20 (0.19)	65.12 (2.26)	7.80 (4.53)
	Salinity	2.00 (0.11)	9.24 (0.72)	59.98 (1.68)	9.04 (1.05)
	Drought	1.86 (0.30)	6.12 (0.23)	55.06 (4.72)	8.98 (2.54)
Piculja	Control	4.24 (0.21)	3.16 (0.53)	67.17 (3.06)	8.02 (1.64)
	Salinity	3.26 (0.22)	1.83 (0.20)	64.28 (1.44)	9.46 (5.76)
	Drought	3.69 (0.05)	1.84 (0.03)	58.00 (1.57)	7.65 (6.34)
PLJ 7	Control	2.37 (0.35)	3.32 (0.10)	78.01 (2.30)	7.32 (1.12)
	Salinity	2.21 (0.20)	3.03 (0.42)	70.91 (6.92)	11.09 (7.50)
	Drought	2.21 (0.16)	4.12 (0.58)	59.79 (2.28)	7.46 (1.08)
PLJ 22	Control	2.93 (0.13)	3.55 (0.27)	75.23 (0.90)	7.87 (2.43)
	Salinity	2.69 (0.36)	3.53 (0.47)	75.47 (5.23)	9.02 (6.83)
	Drought	1.96 (0.36)	3.55 (0.48)	59.80 (1.41)	8.78 (5.90)

Data are presented as averages (SD in parenthesis), n=3

Supplementary Table 4: Mineral Ion Content in Olive Roots

GENOTYPE	TREATMENT	Mg^+ (mg/L)	Ca^+ (mg/L)	K^+ (mg/L)
Koroneiki	Control	2.50 (0.09)*	12.07 (1.03)	84.12 (3.54)
	Salinity	2.47 (0.28)	10.19 (0.62)	70.46 (10.03)
	Drought	3.06 (0.15)	22.30 (1.42)	48.71 (2.21)
Leccino	Control	2.72 (0.09)	10.37 (0.21)	76.14 (2.83)
	Salinity	2.63 (0.59)	10.03 (2.16)	36.90 (0.65)
	Drought	2.51 (0.10)	15.90 (1.05)	38.15 (12.05)
Oblica	Control	2.81 (0.32)	11.67 (1.97)	49.26 (0.54)
	Salinity	2.66 (0.46)	10.35 (2.60)	44.47 (2.00)
	Drought	2.61 (0.03)	14.90 (1.04)	32.57 (1.91)
LA 13	Control	5.82 (1.38)	8.09 (0.26)	61.35 (8.00)
	Salinity	6.18 (0.51)	8.38 (1.24)	43.63 (4.07)
	Drought	6.01 (0.69)	12.32 (4.08)	30.84 (1.88)
LN 11	Control	8.13(1.80)	14.49 (7.64)	61.24 (5.44)
	Salinity	6.89 (2.83)	7.99 (4.06)	48.24 (5.43)
	Drought	6.20 (1.66)	16.30 (2.67)	21.37 (3.42)
PLJ 18	Control	7.70 (0.10)	19.05 (1.12)	39.75 (0.73)
	Salinity	5.48 (0.73)	8.40 (1.73)	22.29 (1.27)
	Drought	8.97 (1.70)	21.43 (3.27)	47.55 (8.71)
MLJ 25	Control	9.55 (0.40)	34.17 (1.64)	74.00 (1.39)
	Salinity	9.05 (0.38)	26.12 (2.32)	46.34 (1.55)
	Drought	9.63 (0.20)	73.64 (10.61)	32.26 (1.89)
Piculja	Control	8.68 (0.40)	32.18 (1.84)	75.51 (3.71)
	Salinity	8.42 (0.57)	27.23 (0.86)	50.41 (0.61)
	Drought	9.73 (0.46)	38.12 (1.30)	35.64 (2.05)
PLJ 7	Control	12.24 (0.55)	33.25 (0.69)	71.34 (3.05)
	Salinity	7.20 (0.68)	23.13 (2.22)	34.59 (2.61)
	Drought	8.19 (1.50)	29.01 (1.57)	43.23 (3.27)
PLJ 22	Control	10.42 (0.82)	25.91 (2.85)	85.85 (6.33)
	Salinity	8.90 (0.69)	21.97 (1.85)	49.28 (5.99)
	Drought	9.26 (0.58)	29.90 (1.22)	37.39 (1.66)

Data are presented as averages (SD in parenthesis), n=3

Supplementary Table 5: Biochemical parameters measured in olive leaves

GENOTYPE	TREATMENT	SOD U/mg protein	GPOD U/mg protein	MDA (nmol/g DM)	Proline (mg/g DM)
Koroneiki	Control	209.71 (10.46)*	0.78 (0.14)	162.65 (7.00)	0.15 (0.02)
	Salinity	131.41 (6.13)	0.40 (0.02)	159.69 (12.36)	0.16 (0.02)
	Drought	142.77 (19.47)	0.53 (0.08)	149.11 (4.23)	0.17 (0.02)
Leccino	Control	1254.18 (157.36)	0.69 (0.03)	128.78 (4.44)	0.27 (0.02)
	Salinity	2201.44 (379.44)	0.25 (0.04)	114.44 (18.97)	0.24 (0.04)
	Drought	2609.35 (283.14)	0.36 (0.04)	115.56 (12.63)	0.28 (0.02)
Oblica	Control	120.30 (4.32)	1.09 (0.10)	159.87 (13.84)	0.18 (0.01)
	Salinity	172.25 (12.00)	0.34 (0.08)	157.07 (11.55)	0.14 (0.03)
	Drought	173.25 (4.00)	0.44 (0.02)	149.20 (16.41)	0.19 (0.07)
LA 13	Control	600.54 (43.12)	1.66 (0.04)	132.64 (17.14)	0.37 (0.02)
	Salinity	426.87 (54.67)	0.41 (0.04)	162.58 (15.90)	0.39 (0.04)
	Drought	1031.86 (60.02)	2.69 (0.32)	121.18 (6.77)	0.41 (0.05)
LN 11	Control	324.36 (3.66)	0.20 (0.03)	101.69 (11.69)	0.30 (0.03)
	Salinity	637.65 (49.68)	0.21 (0.03)	104.64 (7.44)	0.52 (0.05)
	Drought	521.15 (77.11)	0.42 (0.06)	110.84 (15.19)	0.32 (0.10)
PLJ 18	Control	166.89 (1.21)	0.64 (0.06)	105.89 (15.67)	0.25 (0.04)
	Salinity	169.92 (20.75)	0.29 (0.05)	122.67 (24.09)	0.38 (0.05)
	Drought	149.77 (3.14)	0.46 (0.02)	132.66 (26.81)	0.37 (0.06)
MLJ 25	Control	273.52 (4.38)	0.58 (0.04)	141.32 (7.46)	0.14 (0.03)
	Salinity	215.78 (22.70)	0.11 (0.02)	148.88 (31.89)	0.28 (0.04)
	Drought	413.04 (35.49)	0.27 (0.01)	142.47 (23.05)	0.17 (0.03)
Piculja	Control	206.86 (16.51)	0.20 (0.02)	93.88 (11.51)	0.30 (0.01)
	Salinity	205.28 (34.57)	0.49 (0.03)	98.37 (1.64)	0.35 (0.04)
	Drought	258.60 (17.59)	0.43 (0.05)	96.09 (2.09)	0.35 (0.05)
PLJ 7	Control	227.33 (28.03)	0.84 (0.07)	110.37 (18.78)	0.36 (0.02)
	Salinity	123.08 (4.76)	2.03 (0.07)	88.69 (13.43)	0.40 (0.07)
	Drought	428.67 (42.38)	3.18 (0.20)	103.14 (14.91)	0.38 (0.07)
PLJ 22	Control	146.31 (13.16)	1.37 (0.20)	105.71 (14.49)	0.31 (0.05)
	Salinity	265.83 (23.57)	0.58 (0.04)	112.42 (18.85)	0.38 (0.03)
	Drought	255.49 (40.91)	1.24 (0.18)	161.96 (29.24)	0.39 (0.03)

Data are presented as averages (SD in parenthesis), n=3
